# Venous thrombolysis prior to mechanical thrombectomy reduces glycocalyx damage in patients with acute ischemic stroke

**DOI:** 10.3389/fneur.2024.1321909

**Published:** 2024-08-21

**Authors:** Bin Xu, Tengkun Yin, Tanggui Sun, Hang Lv, Wenyv Zhang, Xv Zan, Jiheng Hao, Jiyue Wang, Liyong Zhang

**Affiliations:** ^1^Neurosurgery Department, Liaocheng People's Hospital, Liaocheng, China; ^2^School of Clinical Medicine, Weifang Medical University, Weifang, China; ^3^Joint Laboratory for Translational Medicine Research, Liaocheng People's Hospital, Liaocheng, Shandong, China

**Keywords:** thrombolysis, thrombectomy, glycocalyx, stroke, syndecan-1

## Abstract

**Introduction:**

The administration of intravenous thrombolysis (IVT) before mechanical thrombectomy (MT) in the treatment of acute ischemic stroke (AIS) has been a subject of debate, and its potential benefits remain uncertain. This retrospective study aimed to investigate the effect of preoperative IVT on glycocalyx damage in patients with cerebral ischemia-reperfusion injury (IRI).

**Methods:**

A cohort of 106 patients with acute large vessel occlusion in the anterior circulation treated with mechanical thrombectomy was enrolled. The levels of the glycocalyx damage marker, syndecan-1, were measured in the peripheral blood of these patients to assess glycocalyx damage during IRI, and clinical outcomes were compared between patients receiving MT alone vs. combined IVT and MT.

**Results:**

The study results indicate that thrombolytic drugs have a significant impact on syndecan-1 levels in the blood. Compared to patients who underwent direct MT, those who received preoperative IVT had significantly lower levels of syndecan-1 in their blood. Although preoperative IVT did not alter the final clinical outcomes, the levels of syndecan-1 shedding reflect the extent of damage to the endothelial glycocalyx.

**Discussion:**

This suggests that using thrombolytic drugs before mechanical thrombectomy may reduce endothelial glycocalyx damage in patients with ischemia-reperfusion injury. These findings provide indirect clinical evidence supporting the preoperative use of intravenous thrombolysis in such patients.

## Introduction

Stroke is a significant global health challenge, ranking as the second leading cause of disability and mortality worldwide (1). AIS is the most common form of stroke, accounting for ~87% of all stroke cases. It represents a critical medical emergency characterized by insufficient blood supply to the brain's blood vessels, resulting in damage to brain cells and potentially devastating consequences (2). Timely and effective treatment is crucial in managing AIS to minimize brain injury and improve patient outcomes.

In the management of AIS, IVT and MT are the primary first-line treatment approaches. IVT involves the use of fibrinolytic agents, such as recombinant tissue plasminogen activator (rt-PA), to promote fibrinolysis and dissolve blood clots, causing vessel occlusion. The rt-PA activates plasminogen to plasmin by cleaving the Arg^561^-Val^562^ peptide bond (3). Plasminogen activation to plasmin plays a vital role in degrading fibrin and inhibiting clotting factors, which facilitates thrombus dissolution and restores blood flow (4, 5). Patients with AIS who do not ameliorate after IVT may benefit from transfer to a hospital where MT can be performed, which is known as bridging therapy (6).

MT is a minimally invasive procedure that employs endovascular instruments to directly remove the obstructive thrombus, thereby restoring blood flow in the brain tissue. However, the reestablishment of blood flow after a period of ischemia and hypoxia can lead to IRI, characterized by rapid tissue damage (7). This phenomenon triggers the production of excessive reactive oxygen species and nitrogen in the ischemic brain tissue, promoting the aggregation of pro-inflammatory immune cells at the injury site and causing endothelial glycocalyx dysfunction (8). The endothelial glycocalyx is a protective layer that lines the interior of blood vessels. It is composed of various components, including glycoproteins, proteoglycans such as heparan sulfate proteoglycans (including members of the syndecans family), and glycosaminoglycan side chains. This glycocalyx layer plays a critical role in preserving the integrity and functionality of blood vessels. In the field of stroke, it has also gained increasing attention as a novel marker of cerebral IRI.

Syndecan-1 is a transmembrane proteoglycan that is primarily expressed on the surface of endothelial cells. Under inflammatory and pathological conditions, proteases such as heparanase can cleave the extracellular domain of syndecan-1, causing it to shed into the extracellular matrix and enter the bloodstream. Elevated syndecan-1 levels in peripheral blood during IRI serve as a crucial marker of glycocalyx damage, indicating injury to this protective layer of endothelial cells (9). Our previous study highlighted a significant increase in syndecan-1 shedding during the hyperacute phase of AIS, and its dynamic changes are potentially linked to blood-brain barrier permeability (10). Currently, there is an ongoing controversy within the academic community regarding the effectiveness of bridging therapy with thrombolytic drugs before MT in AIS patients (11–15). However, it is worth noting that this approach is strongly recommended in the guidelines issued by European Stroke Organization (ESO)/European Society for Minimally Invasive Neurological Therapy (ESMINT) (16). The potential benefits of this treatment in preserving glycocalyx integrity and improving clinical outcomes require further investigation and clarification. In this study, our research objective is to observe syndecan-1 levels and evaluate whether preoperative treatment with conventional thrombolytic drugs preserves the integrity of the endothelial glycocalyx after IRI. Understanding the impact of preoperative thrombolytic therapy on glycocalyx preservation and patient prognosis could provide valuable insights for optimizing treatment strategies for IRI.

## Materials and methods

### Sample

Plasma samples were collected from eight healthy individuals aged 50–80 years and 106 AIS patients at Liaocheng People's Hospital between August 2020 and May 2022. Informed consent was obtained from all participants. The inclusion criteria were as follows: (1) age ≥18 years; (2) confirmation of anterior circulation cerebral vessel occlusion through computed tomography (CT) angiography or digital subtraction angiography (DSA); (3) all patients who underwent mechanical thrombectomy (MT) with retrieved stents and received standard medical therapy; (4) patients who received preoperative thrombolytic treatment after being treated with rt-PA; and (5) all patients who achieved successful reperfusion (modified treatment in cerebral infarction score ≥2b). The exclusion criteria included severe inflammatory disease, cancer, autoimmune disease, and cytostatic/immunosuppressive therapy within the past 3 months. Various data, including demographic characteristics, risk factors, occlusion position/cause, time from stroke onset to groin puncture/reperfusion success, stroke severity [National Institutes of Health Stroke Scale (NIHSS)], clinical outcome [modified Rankin Scale (mRS)], intracranial hemorrhage, malignant cerebral edema, and neurological deterioration, were prospectively collected. The NIHSS scores were obtained from patients at admission, 1 day post-operation, 7 days post-operation, and at discharge. Neurological deterioration was defined as an increase in the NIHSS score of ≥4 points during the patient's hospitalization (17).

Ethics approval was obtained from the local ethics committee.

In this retrospective study, due to the unclear association between thrombolytic drugs and syndecan-1 at a specific time point, we initially examined multiple time-point blood samples from 37 patients for a small-sample experiment. The levels of peripheral blood syndecan-1 were measured at different time points during IRI in 37 patients before MT, intraoperatively, and at 1 h, 1, 3, and 7 days after MT. The aim was to investigate the impact of thrombolytic drugs on syndecan-1 levels at various stages of IRI. Based on screening for the most sensitive time point (i.e., 1 h post-operation), the remaining 69 patients were further tested for syndecan-1 levels in peripheral blood at that time point to provide additional clarity to the research results. The total number of samples at this time is 106 (37 + 69).

Intraoperative blood was defined as the extraction of intracranial blood from the distal lesion vessels at the occlusion site using a microcatheter during surgery (18). During our intraoperative collection of distal blood, we relied on the research conducted by Kollikowski AM, which thoroughly delineates the technique for harvesting arterial blood from the core of occluded vessel lumens. For a comprehensive understanding of the procedural steps and accompanying videos, please refer to Kollikowski AM's study on local leukocyte invasion in hyperacute ischemic stroke (18). Before undertaking stent-embolus retrieval in conjunction with the distal aspiration technique, we initiated the procedure with the insertion of micro guidewires and microcatheters, intricately maneuvering the microcatheter through the thrombotic occlusion site to obtain blood samples amid occlusive ischemic conditions. Upon successfully positioning the microcatheter, 1 mm of ischemic blood sample was extracted using a syringe for subsequent laboratory analysis.

All blood samples were centrifuged at 3,000 rpm for 10 min and promptly frozen at −80°C for subsequent analysis.

Syndecan-1 levels were measured using the Human Syndecan-1 ELISA kit (CD138) from Abcam (Cambridge, MA, USA, Cat No. ab46506).

### Statistical analysis

The normality of each variable was tested using the Shapiro-Wilk test. Normally distributed continuous variables were presented as the ±mean standard deviation (SD), while non-normally distributed continuous variables were presented as the median with the interquartile range (IQR). Categorical variables were presented as numbers and percentages. The Student *t*-test or Mann-Whitney *U*-test was used for continuous variables, while the chi-squared test was used for categorical variables. The Wilcoxon rank-sum test was employed to analyze the dynamic changes in syndecan-1 levels at different time points. The primary objective was to compare syndecan-1 levels in the blood between patients who received thrombolytic drugs preoperatively and those who did not, as shown in Table 3. The comparison of syndecan-1 levels between the two groups was conducted using the Mann-Whitney *U*-test. For syndecan-1 levels detected 1 h post-operation, we included confounding factors in the generalized linear model analysis. Furthermore, clinical outcomes between the two groups were compared using odds ratios (OR) or β-coefficients with their 95% confidence intervals, which were analyzed using binary logistic regression models or generalized linear models. Multivariable models were adjusted for potential confounders such as age, sex, and admission NIHSS score.

Statistical analyses were performed using SPSS software (version 26.0), and significant differences were set at a *p*-value of ≤ 0.05.

## Results

**Figure 1:**
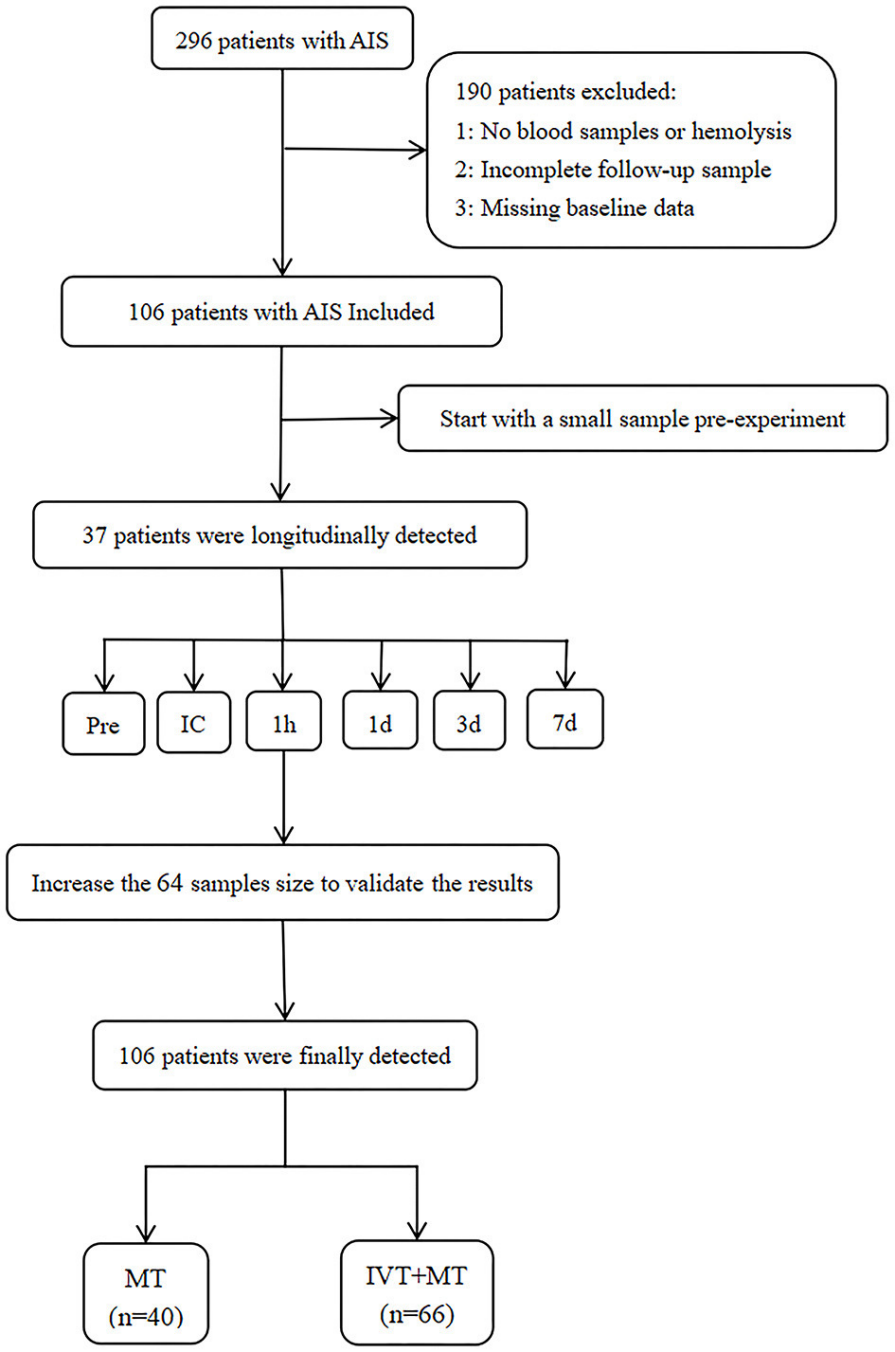
Study flowchart. AIS, Acute ischemic stroke; MT, Mechanical thrombectomy; IVT, Intravenous thrombolysis. Pre, preoperation; IC, intracranial; 1 h and 1 d =1 h and 1 day after MT.

The study included 106 patients, with 66 patients in the IVT+MT group and 40 patients in the direct MT group. A study flowchart is shown in Figure 1. The mean age of the patients was 67 years (SD, ±11), and 74% of the participants were male. The median baseline NIHSS score was 19 points (IQR, 12–21). The median time from stroke onset to groin puncture was 294 min (IQR, 216–401), and the median time from stroke onset to recanalization was 400 min (IQR, 294–538). Among the 16 patients who received thrombolytic therapy at external hospitals, the median time from intravenous administration of thrombolytic drugs to femoral artery puncture was 393 min (IQR, 344–703). Excluding the patients treated at external hospitals, the median time from the intravenous administration of thrombolytic drugs to femoral artery puncture for the remaining patients was 61 min (IQR, 40–109). Although the time from thrombolysis to femoral artery puncture was longer for patients who received thrombolysis at external hospitals than for those who were treated at our hospital, there was no significant difference in syndecan-1 levels between the two groups. The baseline characteristics of the study population are shown in Table 1. The median mRS follow-up score at 90 days was 3 (IQR, 0–5), and the mortality rate within 90 days was 21%. The baseline characteristics between the two groups are compared in Table 2. Baseline characteristics were counterbalanced between the two groups, except for the admission NIHSS score.

**Table 1:** Baseline characteristics of the study population (*n* = 106).

**Sociodemographic characteristics**	
Age, y	67 (11)
Male	78 (74)
**Risk factors**	
Smoking	33 (31)
Drinking	30 (28)
Previous stroke	20 (19)
Hypertension	62 (58)
Coronary artery disease	22 (21)
Atrial fibrillation	36 (34)
Diabetes	10 (19)
Hypercholesterolaemia	17 (16)
**Cause of large-vessel occlusion**	
Cardioembolism	51 (48)
Others/Unknown	55 (52)
**Occlusion position**	
Internal carotid artery occlusion	56 (43)
Middle cerebral artery occlusion	60 (57)
**Clinical metrics**	
Mean systolic blood pressure, mm Hg	153 (±23)
Mean diastolic blood pressure, mm Hg	88 (±12)
Admission NIHSS score	19 (13–29)
Time from stroke onset to groin puncture, min	294 (216–401)
Time from stroke onset to recanalization, min	400 (294–538)

Data are mean (±SD), n (%), median (IQR).

SD, Mean standard deviation; IQR, Interquartile range; NIHSS, National Institutes of Health Stroke Scale.

**Table 2:** Baseline and procedural characteristics of patients treated with MT vs. those treated with IVT + MT.

**Baseline and procedural characteristics**	**MT (*N* = 40)**	**IVT + MT (*N* = 66)**	***P*-value**
**Sociodemographic characteristics**			
Age, y	67 (10)	67 (11)	0.953
**Sex**, ***n*** **(%)**			0.515
Male	28 (70)	50 (76)	
Female	12 (30)	16 (24)	
**Risk factors**, ***n*** **(%)**			
Smoking	13 (33)	20 (30)	0.813
Drinking	14 (35)	16 (24)	0.233
Previous stroke	10 (25)	10 (15)	0.209
Hypertension	20 (50)	42 (64)	0.167
Coronary artery disease	5 (13)	17 (26)	0.103
Atrial fibrillation	15 (38)	21 (32)	0.549
Diabetes	4 (10)	11 (17)	0.505
Hypercholesterolaemia	8 (20)	9 (14)	0.387
**The application of thrombolytic drug**			
External hospital thrombolysis	-	16 (24)	
Median time from thrombolysis to groin puncture, min^*^	-	61 (40–109)	
**Cause of large-vessel occlusion**			0.762
Cardioembolism	20 (50)	31 (47)	
Others/unknown	20 (50)	35 (53)	
**Occlusion position**			0.885
Internal carotid artery occlusion	17 (42)	29 (44)	
Middle cerebral artery occlusion	23 (58)	37 (56)	
**Clinical metrics**			
Mean systolic blood pressure, mmHg	157 (±24)	150 (±22)	0.152
Mean diastolic blood pressure, mmHg	89 (±11)	87 (±12)	0.315
NIHSS score at admission	23 (15–33)	15 (12–22)	0.001
Median time from symptom onset to groin puncture, min	303 (222–523)	282 (208–362)	0.205
Median time from symptom onset to recanalization, min	432 (306–605)	383 (276–504)	0.164

Data are mean (±SD), n (%), and median (IQR).

SD, standard deviation; IQR, Interquartile range; MT, Mechanical thrombectomy; IVT+MT, Intravenous thrombolysis before mechanical thrombectomy; NIHSS, National Institutes of Health Stroke Scale.

^*^Median time from thrombolysis to groin puncture (N = 50): 16 patients who received thrombolytic treatment at external hospitals were excluded due to the inability to determine the exact timing of medication.

**Figure 2:**
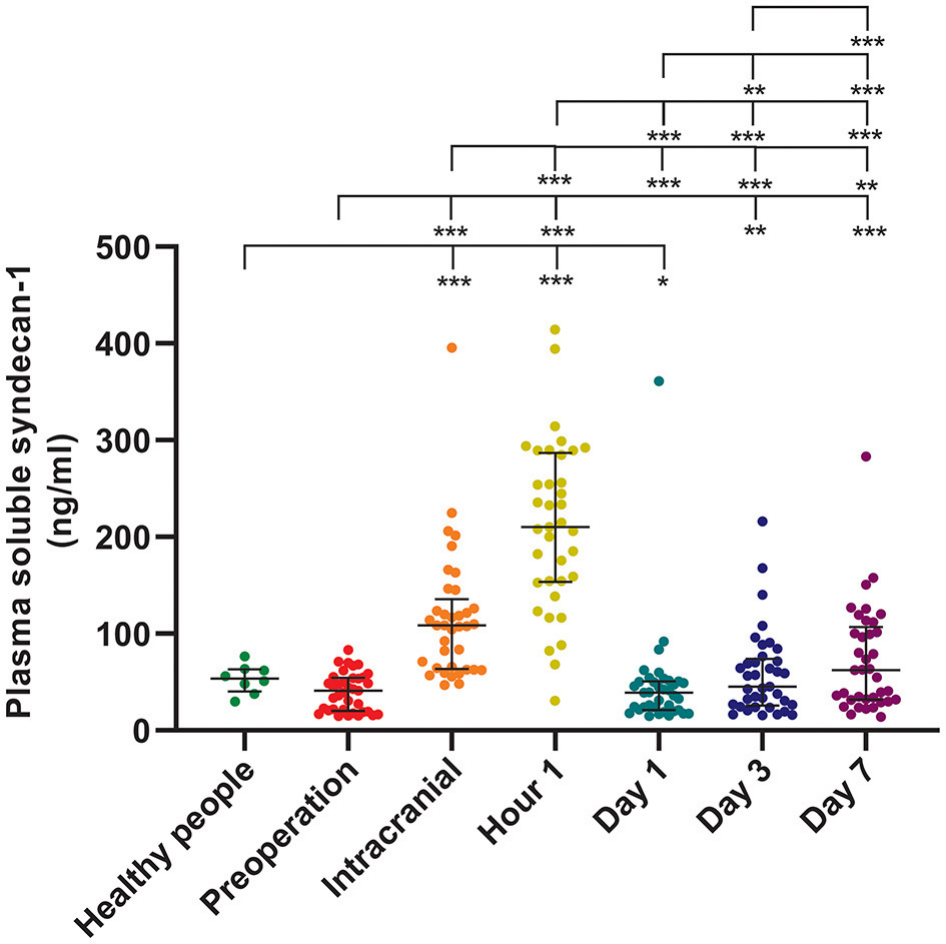
Syndecan-1 in the plasma of healthy individuals and longitudinally dynamics after AIS. Healthy individuals (*n* = 8); AIS patients (*n* = 37); *p < 0.05; **p < 0.01; ***p < 0.001. AIS, Acute ischemic stroke. Data presentation: median (line; 50th percentile) and whiskers (25–75th percentile).

**Figure 3:**
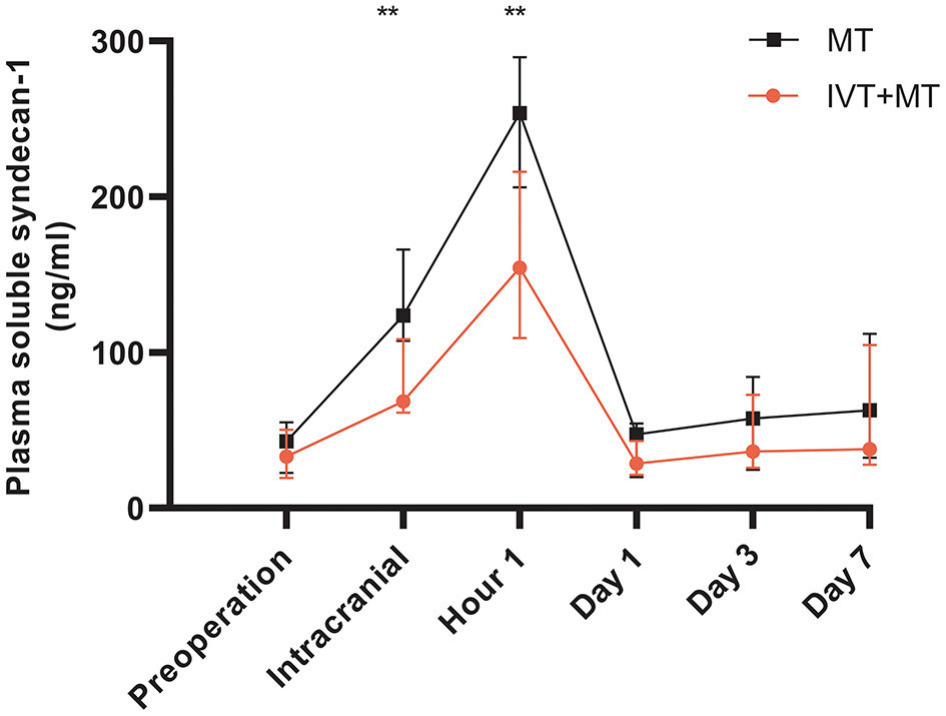
Longitudinally comparison of the MT and IVT+MT. MT, Mechanical thrombectomy (*n* = 19); IVT+MT, Intravenous thrombolysis before mechanical thrombectomy (*n* = 18); IQR, Interquartile range. **P < 0.01. Data presentation: median, IQR (whiskers).

**Figure 4:**
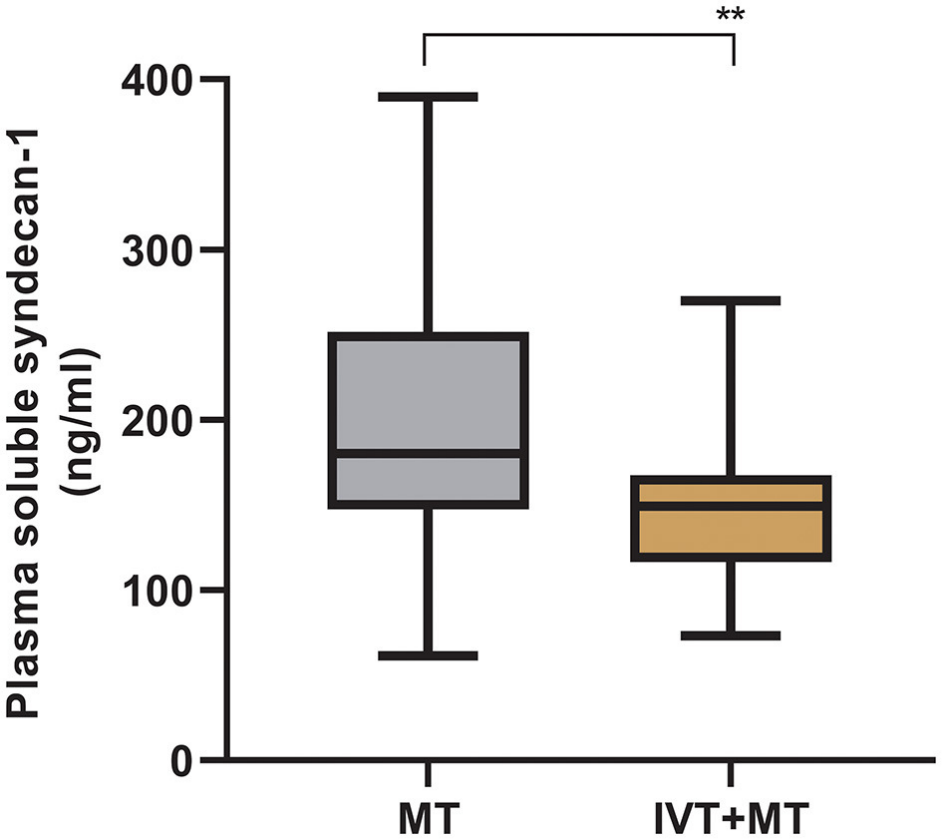
Comparison of syndecan-1 levels between MT and IVT+MT at 1-h post-operation. MT, Mechanical thrombectomy (*n* = 66); IVT+MT, Intravenous thrombolysis before mechanical thrombectomy (*n* = 40), **P < 0.01. The “box” depicts the median and the 25 and 75th quartiles, and the “whisker” shows the 5 and 95th percentile.

The longitudinal assessment of syndecan-1 levels in the peripheral blood of 37 patients at six time points showed a biphasic change after stroke occurrence, with the most significant increase observed at 1 h post-operation. Statistically significant changes were observed at each time point (Figure 2). There were also statistically significant differences in syndecan-1 levels between the two groups only during the intraoperative period and at 1 h post-operation (*p* < 0.01, Figure 3). Specifically, syndecan-1 levels in the blood of AIS patients who received preoperative thrombolysis were lower than those in the direct MT group (Table 3). At 1 h post-operation, the preoperative thrombolysis group showed significantly lower levels of syndecan-1 compared to the direct MT group [median, 149 vs. 180 ng/ml; β = −50.38 [95% CI, −75.54 to −25.22]; *P* < 0.001; Figure 4]. The comparison of clinical outcomes between the two groups of patients is shown in Table 4.

**Table 3:** The syndecan-1 levels of patients treated with MT vs. those treated with IVT + MT.

**Syndecan-1 (ng/ml)**	**MT (*N* = 40)**	**IVT and MT (*N* = 66)**	*P*-value^*^	Adjusted effect size (95% CI)^**^	***P*-value**
1 h post-operation	180 (147–252)	149 (116–167)	0.001	−50.38 (−75.54 to −25.22)	0.000
Longitudinally syndecan-1 (ng/ml)	*N* = 19	*N* = 18			
Pre-operation	43 (23–55)	33 (19–50)	0.395	-	-
Intraoperative	124 (108–166)	68 (61–108)	0.001	-	-
1 h post-operation	254 (206–290)	154 (109–216)	0.001	-	-
1 day post-operation	47 (20–54)	29 (21–43)	0.095	-	-
3 days post-operation	57 (25–84)	36 (26–73)	0.584	-	-
7 days post-operation	63 (32–112)	38 (28–105)	0.584	-	-

Data are median (IQR).

IQR, Interquartile range.

^*^Mann-Whitney U-test.

^**^Adjusted for age, sex, and NIHSS score at admission. The β-coefficients were calculated using a generalized linear model.

**Table 4:** Clinical outcomes of patients treated with MT vs. those treated with IVT + MT.

**Clinical outcomes**	**MT (*N* = 40)**	**IVT + MT (*N* = 66)**	**Effect size (95% CI)**	***P*-value**	Adjusted effect size (95% CI)^*^	***P*-value**
**Primary outcome**					
90-day mRS (0–2)	19 (48)	31 (47)	0.979 (0.446–2.150)	0.958	0.655 (0.265–1.623)^†^	0.361
**Secondary outcomes**					
90-day mRS (0–1)	14 (35)	27 (41)	1.286 (0.570–2.902)	0.545	0.807 (0.317–2.053)^†^	0.652
90-day mRS (0–3)	24 (60)	37 (56)	0.851 (0.383–1.889)	0.691	0.539 (0.214–1.359)^†^	0.190
NIHSS score at 1 day	19 (12–25)	15 (10–23)	−2.646 (-6.471–1.178)	0.175	1.075 (-2.170–4.319)^‡^	0.516
NIHSS score at 7 days	17 (6–25)	12 (6–19)	−3.370 (-7.920–1.179)	0.146	0.405 (-3.581–4.390)^‡^	0.842
Discharge NIHSS score	15 (5–28)	11 (5–19)	−3.344 (-8.252–1.564)	0.182	−0.132 (-4.582–4.318)^‡^	0.954
Discharge mRS (0–2)	13 (33)	21 (32)	0.969 (0.418–2.246)	0.942	0.505 (0.183–1.394)^†^	0.187
**Safety outcomes**					
Death within 90 days	8 (20)	14 (21)	1.077 (0.407–2.852)	0.881	1.539 (0.479–4.947)^†^	0.470
Any ICH within 24 h	11 (28)	18 (27)	0.989 (0.410–2.384)	0.980	1.247 (0.472–3.297)^†^	0.656
Malignant brain edema	10 (25)	11 (17)	0.600 (0.229–1.575)	0.300	0.910 (0.306–2.704)^†^	0.865
Neurological deterioration	10 (25)	22 (33)	1.500 (0.622–3.616)	0.368	1.779 (0.693–4.568)^†^	0.231

Data are median (IQR), n (%).

IQR, Interquartile range; MT, Mechanical thrombectomy; IVT+MT, Intravenous thrombolysis before mechanical thrombectomy; mRS, modified Rankin Scale; NIHSS, National Institutes of Health Stroke Scale; ICH, Intracranial hemorrhage; NIHSS, National Institutes of Health Stroke Scale; OR, odds ratio.

^*^Adjusted for age, sex, and admission NIHSS score.

^†^The OR values were calculated using a binary logistic regression model.

^‡^The β-coefficients were calculated using a generalized linear model.

Among the 106 patients who completed a 90-day follow-up, an adjusted binary logistic regression analysis showed no significant difference in the favorable clinical outcome (mRS score of 0–2) between the two groups [48 vs. 47%; adjusted OR, 0.655 [95% CI, 0.265–1.623]; *P* = 0.361]. There were no significant differences between the two groups regarding secondary and safety outcomes.

## Discussion

In this study, we investigated the effect of preoperative IVT on glycocalyx damage in patients with IRI undergoing MT for AIS. For the first time, we discovered that preoperative thrombolytic drug administration before MT significantly reduced the levels of syndecan-1, a marker of glycocalyx damage, in the blood.

Thrombolytic drugs were found to reduce syndecan-1 levels in the blood, with the most significant effect observed from the intraoperative period to 1 h post-operation. Alteplase exhibits specificity for fibrin, primarily targeting plasminogen and fibrin in the coagulation process, with relatively minimal impact on other biological molecules (pharmacokinetics of alteplase in treating ischemic stroke). The current literature does not explicitly suggest that alteplase directly degrades syndecan-1 after administration. Therefore, the likelihood of alteplase directly degrading syndecan-1 appears low. It may influence syndecan-1 shedding through indirect pathways, particularly impacting the shedding of glycocalyx on endothelial cell surfaces. This finding indicates that thrombolytic drugs may have a certain inhibitory effect on syndecan-1 shedding, thereby preserving the integrity of the endothelial glycocalyx. Thrombolytic drugs achieve their fibrinolytic effect by activating plasminogen to plasmin, which degrades fibrinogen and various coagulation factors such as Factor V (5, 19). Coagulation factor V serves as a cofactor and forms a prothrombinase complex with coagulation factor X, leading to the cleavage of prothrombin during endothelial or cerebral injury, ultimately activating thrombin (20, 21). The activated thrombin then releases heparanase (HPSE) from platelets and granulocytes into the blood by interacting with protease-activated receptor 1 (22).

HPSE is an endoglycosidase that specifically cleaves heparan sulfate chains in the endothelial glycocalyx, leading to the enhanced shedding of syndecan-1 (23, 24). Based on an extensive review of relevant literature, we suggest that thrombolytic drugs may inhibit the shedding of syndecan-1 through the thrombin-HPSE pathway. Existing research has confirmed that syndecan-1 is an important biomarker in response to glycocalyx damage caused by IRI (9). Consequently, thrombolytic drugs may alleviate glycocalyx damage caused by ischemia-reperfusion through the thrombin-HPSE pathway.

Moreover, we found that thrombolytic drugs have a significant effect on syndecan-1 levels from the intraoperative period to 1 h post-operation. However, after more than 24 h post-operation, there were no significant differences in syndecan-1 levels between the two groups. This observation is consistent with the pharmacokinetics of alteplase, which is a thrombolytic drug used in the study (25). In patients who underwent preoperative thrombolytic therapy in our hospital, the preoperative peripheral blood samples were collected ~61 min (40–109) after the administration of thrombolytic drugs. At this time point, syndecan-1 levels in the blood were not affected by the thrombolytic drugs, indicating that the effect of thrombolytic drugs on syndecan-1 was not immediate and could only be observed after a delay of at least 1 h. We compared preoperative syndecan-1 levels with data from healthy individuals and found no significant difference between them. The delayed effect of thrombolytic drugs on syndecan-1 may be because, at the time of preoperative blood collection, syndecan-1 on the surface of the endothelial glycocalyx has not yet undergone significant shedding (26), resulting in the limited observed drug efficacy. It could also simply be due to the fact that thrombolytic agents reduce thrombosis, thereby simplifying the thrombectomy process, alleviating endothelial stress, and resulting in lower syndecan-1 levels in the blood both during and after the procedure.

Furthermore, our study did not reveal any significant clinical benefits in bridging therapy patients when comparing the clinical outcomes between the two groups. While the DIRECT-SAFE (27) and SWIFT DIRECT (15) studies suggest that the combined strategy of IVT before MT is not associated with clinical benefits, the SWIFT DIRECT trial revealed a favorable prognosis rate of 65% in the IVT + MT group compared to the rate of 57% in the group undergoing direct MT. This finding suggests a trend favoring the adoption of a bridging treatment strategy in the SWIFT DIRECT trial, despite previous research indicating that elevated peripheral blood syndecan-1 levels could predict poor outcomes in AIS patients undergoing thrombolytic therapy (28). Our study found no predictive value of syndecan-1 levels in 106 patients for clinical outcomes. The lack of predictive value might be attributed to the inconsistency in the timing of the studies. It is worth noting that syndecan-1 plays a specific role in the pathophysiological processes at various stages of brain tissue injury after stroke. This finding suggests that the dynamics of syndecan-1 levels and their effects on clinical outcomes could be complex and multifactorial. We are conducting a prospective study with a substantial sample size, multiple time points, and an array of markers pertaining to glycocalyx injury. Our primary objective from a clinical perspective is to further validate the impact of thrombolytic drugs on glycocalyx and clinical outcomes in AIS patients. We also seek to confirm the involvement of the thrombin-HPSE pathway, which could shed light on the underlying mechanisms of thrombolytic drug effects on glycocalyx.

However, we must acknowledge certain limitations of this study. First, the sample size and the low proportion of female participants might not be sufficient to ensure the utmost statistical stability and generalizability of the results. There is a significant difference in NIHSS scores upon admission between the mechanical thrombectomy group and the bridging therapy group, which may affect the results. We could not obtain microcirculation vascular imaging data, which hinders our analysis of whether the IVT + MT bridging strategy also facilitates reperfusion of small brain vessels. Therefore, we remain committed to recruiting more patients and incorporating the evaluation of microcirculation vascular imaging data into subsequent clinical data, ensuring the availability of a robust dataset for analysis. Second, practical constraints hindered our ability to perform simultaneous measurements of syndecan-1, HPSE, and coagulation factors in the blood at the same time point for each patient. Addressing this limitation could provide valuable insights into their correlation and validate the thrombin-HPSE pathway. Finally, we recognize that relying solely on syndecan-1 as a single marker of glycan shedding might not offer comprehensive evidence of glycocalyx damage. To address this limitation, we plan to investigate multiple glycocalyx markers and conduct foundational research on relevant pathophysiological mechanisms in the future to further validate the safety and reliability of this conclusion.

## Conclusion

Our study indicates that administering pre-thrombectomy intravenous thrombolytics in AIS patients can effectively reduce the shedding of syndecan-1. This reduction may be attributed to the potential impact of thrombolytic drugs on the thrombin-HPSE pathway, thereby mitigating endothelial glycocalyx damage caused by IRI. This finding may indirectly support bridging therapy and provide a new research perspective on how thrombolytic drugs benefit AIS patients. However, further validation studies will be necessary to gain a deeper understanding of this mechanism and explore its potential therapeutic applications.

## Data Availability

The original contributions presented in the study are included in the article/supplementary material, further inquiries can be directed to the corresponding authors.
